# Tarantulas (Araneae: Theraphosidae) use different adhesive pads complementarily during climbing on smooth surfaces: experimental approach in eight arboreal and burrower species

**DOI:** 10.1242/bio.013144

**Published:** 2015-11-04

**Authors:** Fernando Pérez-Miles, Carlos Perafán, Laura Santamaría

**Affiliations:** Sección Entomología, Facultad de Ciencias, Universidad de la República, Iguá 4225, Montevideo 11400, Uruguay

**Keywords:** New World tarantula, Tarantula climbing, Claw-tufts, Tarsal scopula

## Abstract

Tarantulas are large spiders with adhesive setae on their legs, which enable them to climb on smooth vertical surfaces. The mechanism proposed to explain adhesion in tarantulas is anisotropic friction, where friction is higher when the leg pushes than when it pulls. However, previous studies and measurements of adhesion in theraphosids were performed using dead specimens. To test their ability to climb, we studied static friction of live theraphosid spiders on different surfaces and at different inclines. We compared burrower with arboreal species to test the hypothesis of higher friction in arboreal tarantulas. We found a complementary participation of claw tufts and scopula of anterior and posterior legs when the tarantula climbs. The mechanics of climbing in association with the biological characteristics of the species are discussed.

## Abstract (precis)

**Summary:** We test friction as a proxy of adhesion, comparing arboreal and burrower tarantulas climbing on different surfaces and at different angles.

## INTRODUCTION

Theraphosid tarantulas are usually large-sized spiders which inhabit the ground or trees, in tropical and temperate regions. The most important synapomorphy of this group, together with Barychelids, is the co-occurrence of leg tarsal and metatarsal scopulae and distal claw tufts (Raven, 1985). Scopulae and claw tufts consist of thousands of apically broadened setae that cover ventral surfaces of tarsi and metatarsi as well as the tips of the legs under the paired claws. These setae are oriented at a greater angle to the leg axis than covering setae. Setae of scopulae and claw tufts have a very similar structure but differ in length and density (Foelix et al., 2012; Wolff et al., 2013). They are covered on their distal part by setules with spatula-shaped endings, which increase adhesion. Varenberg et al. (2010) explained that an animal's attachment ability grows with an overall length of the peeling line, which is the sum of the widths of all thin-film elements participating in contact. This was also observed in spiders by Foelix and Chu-Wang (1975), Niederegger and Gorb (2006), Wolff and Gorb (2012a,b), and Wolff et al. (2013).

Adhesive setae enable tarantulas to climb on smooth surfaces, e.g. on leaves and on vertical glass plates (Homann, 1957; Wolff and Gorb, 2015; Lapinski et al., 2015). Foelix et al. (2012) studied the adhesion of some tarantula species on glass and found that arboreal species have better adhesion than terrestrial ones. Several authors (Dunlop, 1995; Foelix, 2011; Foelix et al., 2012; Niederegger, 2013) proposed that when the tarantulas walk on vertical glass they only use claw tufts for adhesion, while tarsal and metatarsal scopulae are used for hunting, to grab and hold on to prey. The use of adhesive setae in prey capture in spiders was first reported by Rovner (1978) and recently studied by Wolff et al. (2013) who proposed the association between the presence of adhesive setae and the condition of hunting without the help of webs.

Niederegger and Gorb (2006) measured the adhesion of scopulae in fresh and dried samples of the theraphosid *Aphonopelma seemanni* and concluded that no adhesion forces occur when the scopula is simply lowered and lifted from a surface, with the leg segments parallel to the surface. Adhesion forces are produced when an axial movement occurs. When the spider leg is pulled, the friction forces are low; however, when the leg is pushed, forces are higher. This adhesion by anisotropic friction was explained by the arrangement of the spatulae; that only face the substrate when pushed in a distal direction. Wolff and Gorb (2013) observed in *Cuppienius salei* that the direction of highest friction is opposite in claw tufts when compared to scopula, but how different pads are used by spiders remains unclear.

The mechanism proposed for adhesion is somewhat inconsistent with our observations in live tarantulas and with the results of Wolff and Gorb (2013), and Wohlfart et al. (2014). When a tarantula captures prey the anterior legs are pulled toward the body, so adhesion would be necessary. However, the predator also needs to be able to get rid of the prey very quickly if it turns out to be too large or too dangerous to be handled. In these cases, the ability to push is important.

When climbing on a vertical or even inverted plane, all the legs are in contact with the surface. However, the exact mechanism of adhesion by anisotropic friction has not been studied in live tarantulas. Considering the morphology of adhesive pads (Wolff and Gorb, 2012a; Wolff et al., 2013) and frictional results for live Araneomorphs (Wohlfart et al., 2014) we hypothesized that: (1) the tip of the tarsi (claw tufts) are involved in pulling adhesion, while scopula are involved in pushing adhesion, and the friction produced by forelegs and hind legs are complementary when the spider climbs vertical surfaces, and (2) higher frictional forces are expected in arboreal species than in burrowers. Consequently, using two surfaces with different frictional properties (glass and Teflon) we predict: (1) higher frictional forces in glass than in Teflon, (2) similar frictional forces in mixed surfaces (glass in the upper half and Teflon in the lower half, and vice versa), (3) higher frictional forces in arboreal species than in burrowers.

## RESULTS

### Comparison between species and sexes

**Fig. 1.**
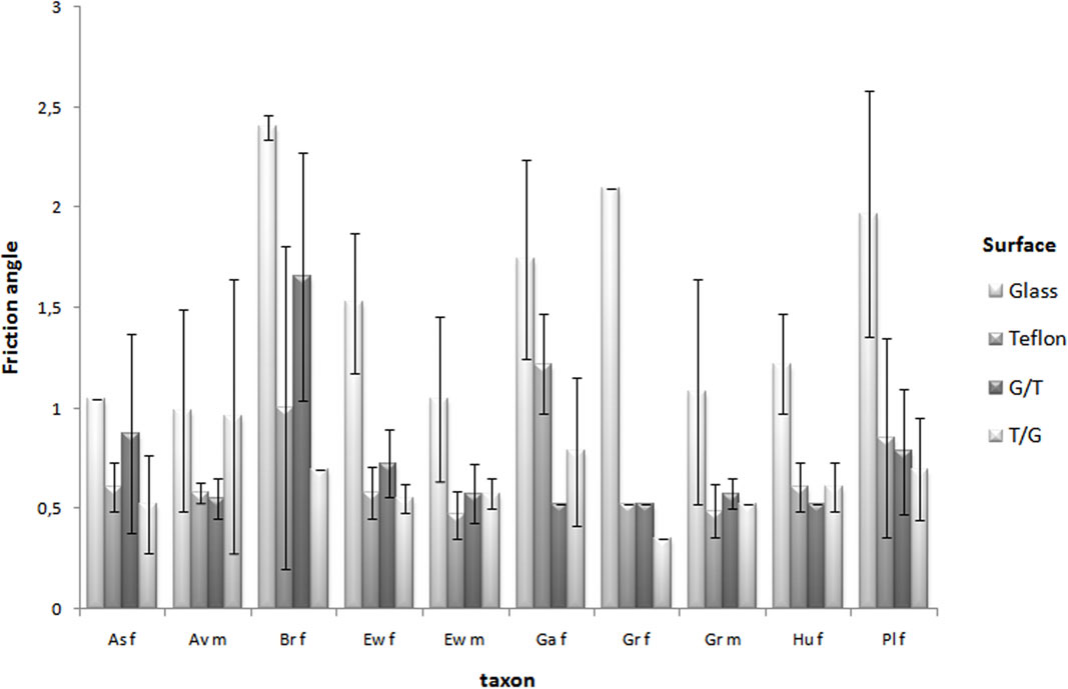
**Means and standard deviations of static friction angle by species and sexes in four surfaces.** As, *Aphonopelma seemanni*; Av, *Avicularia* sp.; Br, *Brachypelma* sp*.*; Ew, *Eupalaestrus weijenberghi*; Ga, *Grammostola anthracina*; Gr, *Grammostola* sp; Hu, *Homoeomma uruguayense*; Pl, *Plesiopelma longisternale*; f, females; m, males; G/T, glass/teflon; T/G, teflon/glass.

We did not find significant differences in friction among species for any of the four surfaces we tested (Table 1, Fig. 1). Furthermore, when we performed pairwise comparisons of species represented by males we did not find significant differences between the arboreal *Avicularia* sp. and the burrowers *E. weijenberghi* (t=0.18, *P*=0.86) and *Grammostola* sp. (t=0.24, *P*=0.82).

**Table 1.**
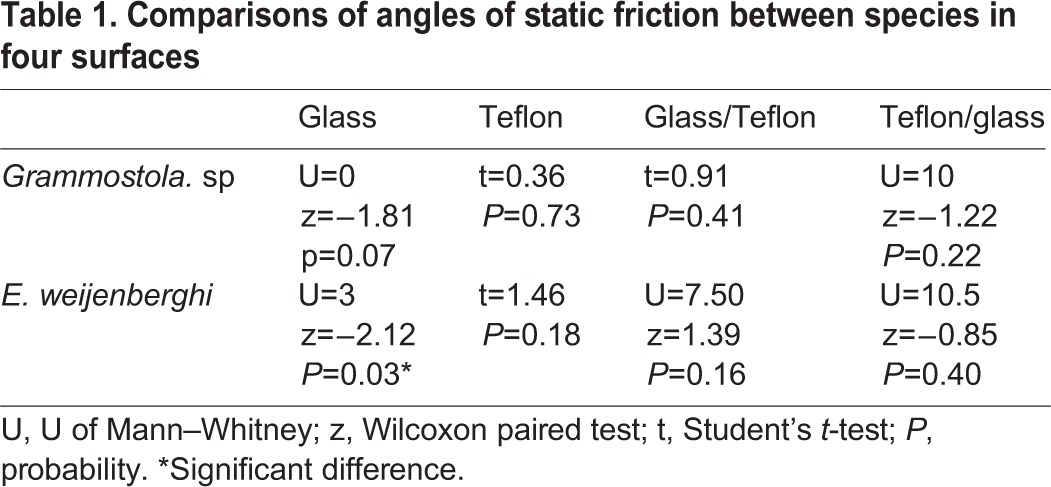
**Comparisons of angles of static friction between species in four surfaces**

When we compared the friction between males and females, in the species with both sexes represented, the only significant difference was for *Eupalaestrus weijenberghi* on glass (Table 2).

**Table 2.**
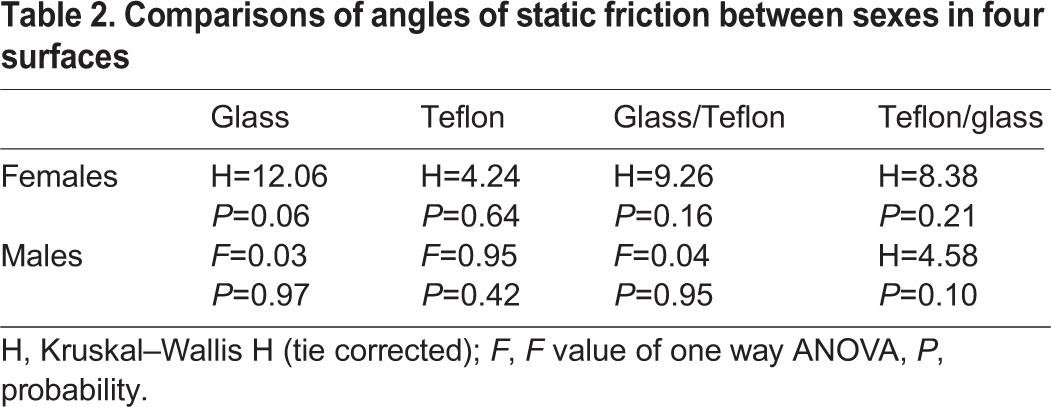
**Comparisons of angles of static friction between sexes in four surfaces**

### Comparisons between surfaces

**Fig. 2.**
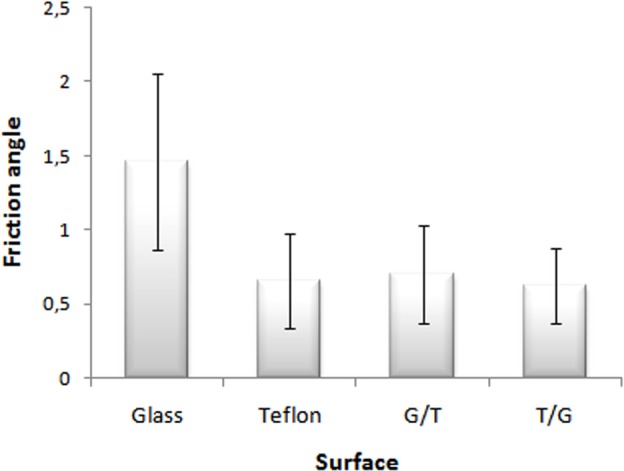
**Mean and standard deviations of all individuals in different surfaces.** G/T, glass/teflon; T/G, teflon/glass.

The individuals of all species showed higher friction on glass than on Teflon (Wilcoxon paired test Z=5.01, *P*=0.00000005, Fig. 2). This result allowed us to use mixed surfaces in order to test the frictional differences between forelegs and hind legs. We did not find significant differences between glass-Teflon and Teflon-glass surfaces in any of the species (Z=1.35, *P*=0.18). Considering all individuals together, we did not find a significant correlation between weight and adhesion on glass (r=0.32, *P*=0.13), teflon (r=−0.05, *P*=0.81) glass/teflon (r=−0.07, *P*=0.76) and teflon/glass (r=−0.22, *P*=0.32).

### Observations about climbing

**Fig. 3.**
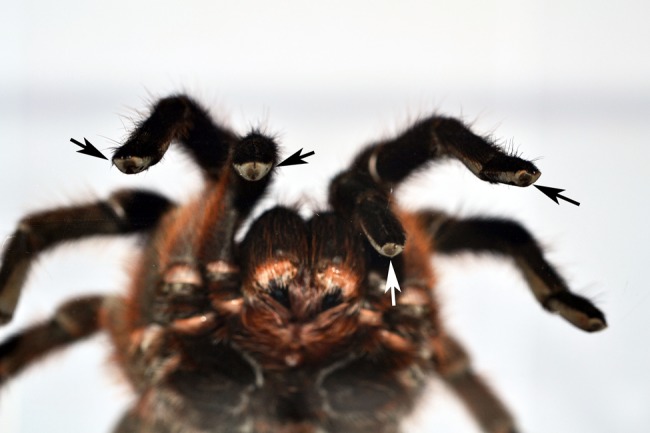
**Ventral view of the contact surface of palps and forelegs of a female *G. anthracina* climbing upwards on a vertical surface of glass.** Mainly claw tufts make contact with the glass (arrows).

**Fig. 4.**
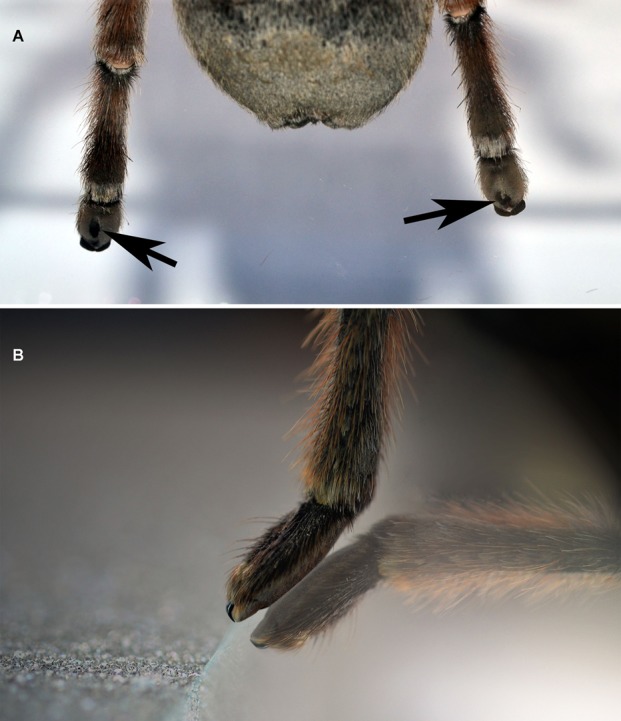
**Female *Grammostola anthracina* climbing upwards on a surface of glass.** (A) Ventral view of the contact surface of leg IV on a vertical surface. Mainly the distal half of tarsal scopula is contacting the glass (arrows). (B). Lateral view of the contact surface of leg IV on a inclined surface of glass. Only part of the tarsal scopula contact with the surface.

During climbing upwards, palps, legs I and II touched the surfaces only with part of the claw tufts (Fig. 3), while leg pair IV touched the surface with the distal portion of tarsal scopulae and only in rare cases with claw tufts (Fig. 4A,B). Leg IV was more extended than the resting legs. This pattern was observed in all species on glass as well as on Teflon. When climbing on, legs III usually touched the surface with part of the claw tufts but in some cases, when this leg was extended backwards, it also touched the surface with the distal portion of the tarsal scopulae. During increased inclination the contact area of anterior claw tuft increased as well as the posterior scopula, while the contact area of posterior claw tufts slightly decreases. Usually, just before a leg is elevated for a step, we observed movements in the tarsal claw tufts.

## DISCUSSION

According to our results, the differences in extension of scopulae among species and the condition of scopula (entire or divided) did not show consequences on friction abilities. Foelix et al. (2012) found higher adhesion in the African arboreal tarantula *Poecilotheria ornata* when compared to the ground species *Brachypelma smithi*. *Avicularia* sp. is the only arboreal species used in this study; it has very extended and conspicuous scopulae on the tarsi but unexpectedly did not show differences of friction when compared to terrestrial species on any surface. The scopulae in theraphosids can form one entire field or be divided by a longitudinal band of longer conical setae (Pérez-Miles, 1994; Guadanucci, 2005). Two species included in this study have divided scopulae: *H. uruguayense* and *P. longisternale;* but we did not find differences in friction in comparison with the other studied species; although, conical longer setae of the middle scopula line was not interpreted as adhesive setae (Pérez-Miles, 1994). The absence of correlation between the weight and the friction values in the studied species could be explained because the extension of adhesive pads is probably proportional to spider size (and weight), compensating for the differences in weight.

Males of *E. weijenberghi* had significantly lower friction than females on glass. This result could be explained by the conspicuous sexual differences in the biology of adults. Males live approximately two months as adults, and after maturation molt considerably increase their locomotion to search for females, while females live several years and are strictly burrowers (Pérez-Miles et al., 2005, 2007). The intense locomotion of males produces body deterioration (Pérez-Miles et al., 2005) and consequently wastage of adhesive pads reducing friction values.

The higher friction on glass than on Teflon, observed for all studied species, contrasts with the high adhesion to Teflon found by Roscoe and Walker (1991) in *Salticus scenicus*. The differences in friction found here allowed us to use mixed surfaces to test whether an anisotropic friction was responsible of the adhesion as proposed by Niederegger and Gorb (2006) or the alternatives indicated by Wolff and Gorb (2013) and Wohlfart et al. (2014). Since species show more adhesion on glass than on Teflon and considering that Niederegger and Gorb (2006) proposed that legs produce more adhesion when pushing compared to pulling, a higher adhesion was expected when the inferior half of the substrate was glass. In this situation, posterior legs that push would be on glass (good substrate for friction) and forelegs that pull would be on Teflon. Conversely, when Teflon was on the inferior half, the legs that push are on this surface with low friction, so lower friction was presumed. No differences were observed between both mixed surfaces and the main friction of forelegs or hind legs is enough to ensure climbing in tarantulas. These results agree with Wohlfart et al. (2014) who found complementary adhesion in *Cupiennius salei* on glass disabling anterior and posterior legs.

Our findings partially agree with the morpho-functional explanation given for both scopulae and claw tufts by Niederegger and Gorb (2006). These authors propose that the scopulae or claw tuft setae are curved in the proximal direction when pushed. In our observations, scopulae made contact with the substrate when the leg pushed, while claw tufts made contact when the leg pulled or during locomotion on a horizontal plane. Based on this observation and considering that the morphology of scopulae and claw tufts may function in a different way: scopulae produce adhesion when the leg pushes while claw tufts produce adhesion when the leg pulls. This observation is congruent with the opposite direction in friction forces of scopula and claw tufts found in *Cuppienius salei* and *Aphonopelma seemanni* (Niederegger and Gorb, 2006; Wolff and Gorb, 2013). These results could be explained by the opposite arrangement of adhesive setules, which are present on the dorsal (facing substrate) part of scopula setae, as reported by Foelix and Chu-Wang (1975) and Niederegger and Gorb (2006), and on the ventral (facing body) part (Hill, 2010; Wolff and Gorb, 2012a,b, 2013) of claw tuft setae. In agreement, our unpublished preliminary observations with SEM in Theraphosids, confirm the presence of setules on the proximal part of claw tufts setae. Foelix et al. (2012) suggested that adhesive setules are on opposite faces on the claw tufts and tarsal scopula setae, but this author proposed different arrangement for the setules (ventral for scopulae and dorsal for claw tufts). Wolff and Gorb (2013) also found that the orientation of setae gradually changes in distal and lateral directions of claw tufts and scopula, which could influence the direction of friction force in relation to the part of the leg in contact with the substrate. This factor could optimize the complementary friction of claw tufts and scopulae including lateral components of leg movement.

Most authors attributed the locomotory adhesive function to claw tufts and adhesion for prey capture to tarsal scopula (Rovner, 1978, 1980; Dunlop, 1995; Foelix, 2011; Pekár et al., 2011; Foelix et al., 2012; Niederegger, 2013; Wolff and Gorb, 2012a, 2015; Wolff et al., 2013; Lapinski et al., 2015). However, we found that at least scopulae of tarsi IV touched the surface while climbing on vertical surfaces, which suggests that tarsal scopulae could also participate in locomotory adhesion. Additionally to locomotory function, we found that claw tufts produce adhesion when a leg pulls which suggest the use of claw tufts of forelegs for prey capture. In general terms, anisotropic friction could explain the adhesion during locomotion but in different ways for scopulae and claw tufts. The scopula produces adhesion when the leg pushes mainly for locomotion, especially when climbing on inclined surfaces, and claw tufts produce adhesion when the leg pulls using this adhesion for locomotion but also in forelegs for prey capture.

The question remains: why do tarantulas with 80% of the species terrestrial and burrowers have such effective adhesion mechanisms? One possibility would be that ancestral species lived in trees and then conquered terrestrial habitats so they retain the adhesive pads that early evolved for climbing. This hypothesis however, is in conflict with the known phylogenies of Theraphosidae in which the arboreal condition was acquired several times independently (West et al., 2008; Bertani, 2012). A costly feature such as having scopulae is reduced when it is not needed, hence there must be a benefit. Interestingly, scopulae and claw tufts are also present in other burrowing mygalomorphs, such as the Nemesiidae or Barychelidae. The distal scopulae and claw tufts could play a significant role in prey capture and prey fixation. Maybe the improved climbing ability is just a side effect, and an important precursor that made the occupation of above ground habitats possible, as in arboreal spiders. This is consistent with the interpretation of Wolff et al. (2013) and Wolff and Gorb (2015), that the primary adaptive value of the adhesive pads was prey capture. The fact that they are, important climbing tools are secondary characters that evolved with the occupation of above ground habitats. Solifuges which are strictly ground living arachnids have adhesive pads in their pedipalps that are used in prey capture (Willemart et al., 2011). These can also be used to climb steep smooth surfaces, but it is obvious that this is not the primary function of the pads (Cushing et al., 2005).

## MATERIALS AND METHODS

We used 35 individuals of eight species of theraphosids (one arboreal and the rest terrestrial species). Two females of *Aphonopelma seemanni* from Torre Molinos, Guatemala, 15 Aug. 2008, Ortiz; three males of the arboreal *Avicularia avicularia* from Pará, Belem, Brazil, Jul. 2013; two females of *Brachypelma* sp. from San Pedro, Guatemala, Jun. 2008, Ortiz; five females of *Eupalaestrus weijenberghi* from Paysandú, Uruguay, 2010-2011, Perdomo; one female from Montevideo, Uruguay, 28 Oct. 2009, Useta, and five males from Canelones, Uruguay, 2 Mar. 2013, Costa; two females and five males of *Grammostola* sp., from Durazno, Salto and Tacuarembó, Uruguay, 2001-2013; two females of *Grammostola anthracina* from Colonia and Lavalleja, Uruguay, 2008-2011; two females of *Homoeomma uruguayense* from Maldonado and Florida, Uruguay, 1 Jun. 2010, 30 Apr. 2013; five females of *Plesiopelma longisternale* from Rivera, Uruguay, Sep. 2008 and one female from Canelones, Uruguay, 5 Jul. 2004, Aisenberg.

We included tarantulas with different lifestyles and scopula conditions: the arboreal *Avicularia* sp. live in tropical forests of South America, all other studied species are burrowers. *Grammostola* spp., *Plesiopelma longisternale,* and *Homoeoma uruguayense* inhabit the underside of rocks, in stony hills of the Pampean Province. *P. longisternale* and *H. uruguayense* are the only species with divided scopulae. *Eupalaestrus weijenberghi* live in Pampean meadows; *Brachypelma* sp. and *Aphonopelma seemanni* in tropical forests of Central and Southern North America.

We maintained individuals under laboratory conditions, in 25×15 cm and 20 cm high glass terraria with a layer of soil. They were fed weekly with cockroaches (*Blaptica dubia*). The temperature in the laboratory varied between 19.0 and 26.7°C (22.73±2.32) and relative humidity between 60 and 65%. The care and use of experimental animals in this study met with all relevant animal welfare laws, guidelines and local policies.

**Fig. 5.**
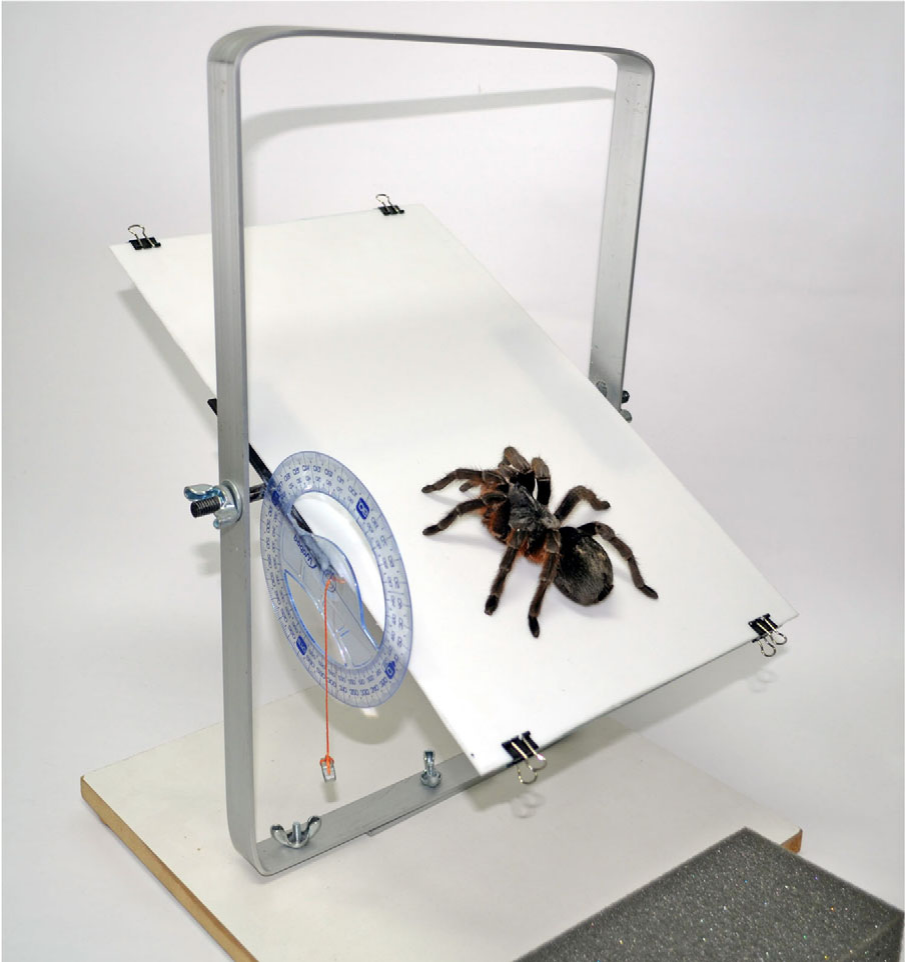
**Experimental apparatus with a rotatory platform and a goniometer to measure the angles.** Surfaces of the platform can be changed.

To test friction on different surfaces and inclinations we used a gyratory platform (Fig. 5). Tarantulas were placed in a horizontal position with the anterior oriented towards the side we would elevate. We tilted the platform slowly (10° per second) until the spider slipped. At this point, we measured the angle of the platform (angle of static friction). We used four different surfaces, two integral: glass and Teflon, and two mixed. The first mixed surface (glass-Teflon) had half glass (on the upper half) and half Teflon (on the lower half). The second mixed surface (Teflon-glass), had half Teflon (on the upper half) and half glass (on the lower half). When we used mixed surfaces, the spiders were placed with the forelegs on one half and the hind legs on the other half. A foam rubber sheet was placed at the bottom to prevent the spider from falling. Each spider was exposed to the four surfaces in different sequences to avoid the possibility of learning. The same individual was reused usually within a week or a minimum interval of 24 h (in *Avicularia* sp.). We weighed every individual to 0.1 mg precision after each observation. We recorded all observations on video. Additional observations and video recording of tarantulas climbing upward were made using a Zoomy handheld digital microscope through a vertical glass.

We compared the angles of static friction (the angle of the platform in the moment the spiders starts to slip, which represents the maximum force), converted from degrees to radians. For statistical comparisons of species and sexes we tested for normality (Shapiro–Wilk W) and homogeneity of variances (Levene test) and, consequently, we used a parametric test (Student's *t*-test and one way ANOVA) or non-parametric test (Kruskal–Wallis and Mann–Whitney tests). To compare friction between different surfaces, individual results were aligned using paired Wilcoxon test. We analyzed the linear correlation between weight and friction (slipping angle in radians) with log transformation for variables, in all individuals together. We used Past version 2.17b package (Hammer et al., 2001). Abbreviations: H, Kruskal–Wallis H tie corrected; *F*, *F* value of ANOVA; *P*, probability; U, U of Mann–Whitney.
